# Suicidal behaviors in depressed adolescents: role of perceived relationships in the family

**DOI:** 10.1186/1753-2000-7-8

**Published:** 2013-03-16

**Authors:** Angèle Consoli, Hugo Peyre, Mario Speranza, Christine Hassler, Bruno Falissard, Evelyne Touchette, David Cohen, Marie-Rose Moro, Anne Révah-Lévy

**Affiliations:** 1 Department of Child and Adolescent Psychiatry, GH Pitié-Salpêtrière, APHP, Paris, F-75013, France; 2 Centre de Soins Psychothérapeutiques de Transition pour Adolescents, Hôpital d’Argenteuil, Argenteuil, Argenteuil, France; 3 Department of Child and Adolescent Psychiatry, Centre Hospitalier de Versailles, Le Chesnay, France; 4 Maison de Solenn, Hôpital Cochin, APHP, Paris, F-75014, France; 5 Département de Santé Publique, Hôpital Paul Brousse, APHP, Villejuif, F-94804, France; 6 INSERM U-669, PSIGIAM, Paris, F-75679, France; 7 Univ. Paris-Sud, Univ. Paris-Descartes, Paris, F-75005, France; 8 Research Unit on Children’s Psychosocial Maladjustment, University of Montreal, Montreal, Canada; 9 CNRS UMR 7222, Institut des Systèmes Intelligents et Robotiques, University Pierre et Marie Curie, Paris, France

**Keywords:** Suicide, Depression, Adolescent, Community survey

## Abstract

**Context:**

Suicide is the second leading cause of death in adolescents and young adults in Europe. Reducing suicides is therefore a key public health target. Previous studies have shown associations between suicidal behaviors, depression and family factors.

**Objective:**

To assess the role of family factors in depression and suicidality in a large community-based sample of adolescents and to explore specific contributions (e.g. mother vs. father; conflict vs. no conflict; separation vs. no separation) taking into account other risk factors.

**Methods:**

A cross-sectional sample of adolescents aged 17 years was recruited in 2008. 36,757 French adolescents (18,593 girls and 18,164 boys) completed a questionnaire including socio-demographic characteristics, drug use, family variables, suicidal ideations and attempts. Current depression was assessed with the Adolescent Depression Rating Scale (ADRS). Adolescents were divided into 4 groups according to suicide risk severity (grade 1 = depressed without suicidal ideation and without suicide attempts, grade 2 = depressed with suicidal ideations and grade 3 = depressed with suicide attempts; grade 0 = control group). Multivariate regressions were applied to assess the Odds Ratio of potential risk factors comparing grade 1, 2 or 3 risk with grade 0.

**Results:**

7.5% of adolescents (10.4% among girls vs. 4.5% among boys) had ADRS scores compatible with depression; 16.2% reported suicidal ideations in the past 12 months and 8.2% reported lifetime suicide attempts. Repeating a year in school was significantly associated to severity grade of suicide risk (1 and 3), as well as all substance use, tobacco use (severity grades 2 and 3) and marijuana use (severity grade 3), for girls and boys. After adjustment, negative relationships with either or both parents, and parents living together but with a negative relationship were significantly associated with suicide risk and/or depression in both genders (all risk grades), and Odds Ratios increased according to risk severity grade.

**Conclusion:**

Family discord and negative relationship with parents were associated with an increased suicide risk in depressed adolescents. So it appears essential to take intrafamilial relationships into account in depressed adolescents to prevent suicidal behaviours.

## Background

Suicide is the third leading cause of death in adolescents and young adults in the United States and the second leading cause in European countries [1]. Suicidal behaviors are also the most common reason for adolescent psychiatric hospitalizations in many countries [2]. Reducing suicide and suicide attempts is therefore a key public health target. In the United States, the death rate by suicide is 6.9/100 000 in adolescents aged 15 to 19 [3]. In France, recent epidemiological data showed that the suicide rate in adolescents aged 15 to 19 is 4.1/100 000 inhabitants [4]. Considerable variability exists among the European countries that published their statistics regarding death rates by suicide in 2008 [5]. Prevalence of suicidal ideations ranges from 15 to 25% in the general population, whereas the lifetime estimates of suicide attempts among adolescents range from 1.3 to 3.8% in males and from 1.5 to 10.1% in females, with higher rates in females than in males in the older age range [6].

Current models of suicide phenomena in adolescents emphasize: (i) the importance of distinguishing suicidal ideation, non-suicidal self-harm, suicide attempt and completed suicide [7,8] (ii) the key role of depression in the transition from suicidal ideations to suicide attempts, in which depression is a strong proximal factor [9]; (iii) the fact that the numerous risk factors identified do not capture the whole risk leading to the idea that protective factors should be taken into account for suicide risk prediction [10]. Risk factors for completed and attempted suicide have been widely studied. First, psychiatric disorders are present in about 90% of suicidal adolescents [6]. Depressive disorders are consistently the most prevalent psychiatric disorder among adolescents who commit suicide with a prevalence ranging from 49% to 64% and among adolescents who attempt suicide [6,11,12]. Secondly, adolescents who attempted suicide in the past are up to 60 times more likely to commit suicide than those who have not [6]. Also, self-harm is an important predictor of future completed suicide [13]. Thirdly, substance abuse plays a significant role in adolescent suicide and in suicide attempts, especially in older adolescent males when it is comorbid with mood disorders or disruptive disorders [14,15]. Fourthly, social factors such as socio-economic status, school exclusion and social isolation have been also implicated [16,17]. Finally, several studies pointed a significant association with family factors, including family psychopathology, abuse, loss of a parent (death, divorce), intrafamilial relationships, familial cohesion, support and suicidality [16,18-20].

Indeed, the family factors, and especially the perceived quality of family relationships, have been pinpointed as an important risk or protective factor in clinical and community samples of adolescents [1,2,6,21-26]. However, only few population-based studies have examined family factors [19]. They showed several predictive or associated factors, like: poor family environment (low satisfaction with support, communication, leisure time, low parental monitoring) [27], low family support [28], low family cohesion [29], poor family functioning, poor parent–child attachment and problems of parental adjustment [1,19]. On the contrary, higher family cohesion has been reported as a protective factor against future suicide attempt [26] as well as having positive relationships with a parent [30,31]. Improved family connectedness was related to less severe depressive symptoms and suicidal ideation [32]. Nevertheless, equivocal findings exist with regard to the relationship between adolescents’ suicidal behaviours and family variables. This is mainly due to methodological limitations, such as considering only parental marital status (e.g. [22]) or parents together (e.g. [33]), and ignoring other common risk factors from multivariate analysis (e.g. [16,19,34]). Moreover, data suggest a different effect of family factors on suicidal behaviours according to gender (e.g. [34]), clinical severity (e.g. [34]), parental marital status (e.g. [22]), dissatisfaction with relationship with parents (e.g. [33]), and different relationship with mother vs. father (e.g. [34]).

Notwithstanding these interesting results, the complex association between family factors, depression and suicidal behaviors among adolescents remains to be explored in samples large enough to allow multivariate analysis, so as to understand specific contributions (e.g. mother vs. father; conflict vs. no conflict; separation vs. no separation) taking into account other risk factors and severity of depression and suicidal behaviors. The aim of the present study was to assess the link between family factors and suicidal behaviors, adjusting for several potential confounding factors, in a large community-based sample of adolescents aged 17 years. Given that the prevalence of suicide differs substantially between boys and girls, we hypothesized that the impact of familial risk factors would differ according to gender. Similarly, given the role of current depression, we hypothesized that family risk would be related to depression severity, defined as depression associated with suicidal ideation in the last year and/or life-time suicide attempt.

## Methods

### Participants

**Figure 1:**
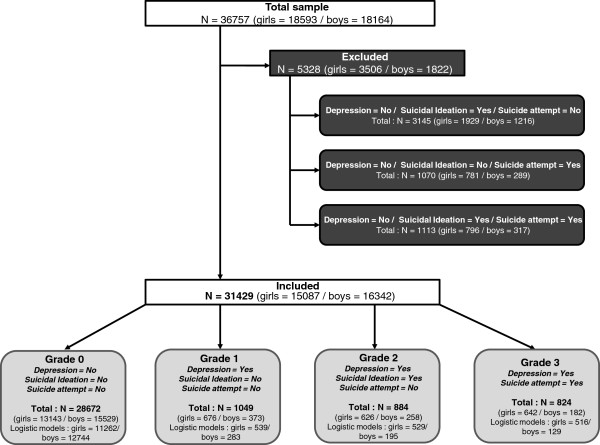
Flowchart.

Participants were recruited in a representative sample of young people from metropolitan France (i.e. all European parts of France, excluding overseas territories) between March 15th and March 31st 2008 during the National Defense Preparation Day “*Journée d’Appel de Préparation à la Défense*” (JAPD) [35]*.* The JAPD is a civic and military information session that is required of all adolescents aged 17, and required to sit public examinations (e.g., driving license, university exams). All 764,000 French adolescents aged 17 and living in metropolitan France in 2008 are called to participate in these national days in one of the 250 Centers [36]. Two days were randomly selected during which all adolescents (n = 44, 733, 5.9%) were invited to participate anonymously in the Survey on Health and Behaviour: “Enquête sur la Santé et les Consommations lors de l’Appel de Préparation A la Défense” (ESCAPAD) [35,37], a cross-sectional survey conducted by the French Monitoring Center for Drugs and Drug Addictions or ”*Observatoire Français des Drogues et des Toxicomanies*” (OFDT), and administered during JAPD days in collaboration with the Army National Service Office. The participation rate for this survey was 88.4%. Thus, the total sample included 36,757 French subjects living in metropolitan France (n = 18,590 girls and 18,163 boys). This represents 4.8% of adolescents aged 17 living in metropolitan France. Among the total sample, we excluded adolescents without current depression but presenting suicidal ideations or a history of suicide attempts (n = 5,328). We excluded these subjects because we were interested in studying the role of current depression as a proximal variable of suicidality and its association with familial risk factors. Our sample finally included n = 31,429 adolescents (see flowchart in Figure 1). The same analyses conducted in this study were additionally performed on the excluded sample, and showed similar results for family risk factor (see Additional file 1: Figure S1). The survey obtained the public statistics general interest and statistical quality seal from “*Comité National de l’Information Statistique*” (CNIS) as well as the approval of ethics committee.

### Assessment

The ESCAPAD survey is a self-administered questionnaire which takes 35 minutes to complete. The response rates for socio-demographic characteristics, familial variables, suicidal behaviors and potential confounding factors were higher than 90%.

#### Depression, suicidal ideation, and suicide attempts

Current depression was assessed using the “Adolescent Depression Rating Scale” (ADRS), specifically developed to assess depression intensity among adolescents. This scale has been previously validated on young people aged 12 to 20 and published with an official cut-off [38]. It is a 10-item self-administered questionnaire with yes/no responses concerning the two weeks preceding completion. The sum of item scores provides a score that divides the population into three distinct groups: score 0 to 2 “not depressed”, 3 to 5 “sub-threshold depression”, and 6 or more “depressed”. The cut-off of 6 was chosen because it provides maximum sensitivity and specificity in screening for major depressive states according to DSM-IV with clinically relevant intensity corresponding to a CGI score (Clinical Global Impression) of 5 or more (i.e. markedly ill or more) [38].

Suicidal ideations were measured by one question: “During the past 12 months, have you had suicidal thoughts?” Responses to this question were never, once, and several times. Suicide attempts (SA) were also explored by one question: “Have you ever tried to kill yourself?” Responses to this question were: never, once, and several times. For the aim of our study, three groups were defined on the basis of the association between depression (i.e. score > 6 on ADRS) and levels of suicidal severity, as follows: grade 1: depressed without suicidal ideation and without suicidal attempt, grade 2: depressed with suicidal ideations and grade 3: depressed with suicide attempts. The control group included adolescents with none of these problems (i.e. < 6 ADRS, no suicidal ideations nor suicide attempts).

#### Family factors

Parental status was measured by the question: “Do your parents live together?” answered by yes or no. Parental harmony had four categorical levels: 1) Living together and good agreement, 2) Separated and discord, 3) Separated and good agreement, and 4) Living together and discord, and was measured by the combination of two questions: 1) “Do your parents live together?” answered by yes or no and 2) “How your parents get along?” with responses scored on a 4-point Likert scale which were dichotomized to increase the clinical relevance of results (i.e.“very well, well, and fairly well” and “badly, and very badly”). The quality of the perceived relationship with the mother and with the father was assessed by the questions: “How do you get along with your mother?” and “How do you get along with your father?” on the same Likert scales and with the same dichotomization as the previous variable. Cohabitation status was measured by a yes or no answer to the question: “Do you live with your parents most of the time?”

#### Potential confounding variables

The following covariates were included because of their potential association with depression, suicidal ideations and suicidal behaviors in adolescence. First, the adolescent’s educational level was assessed in three categories: 1) normal high school attainment, 2) vocational school or apprenticeship and 3) out of school. Secondly, repeat school years were explored via a specific question (it can be noted that it is a more frequent practice in France than in the US and other European countries). Thirdly, socio-economic status (SES) was based on the higher occupational category of the two parents reported by the adolescent, based on the typology of the National institute for statistics and economic studies [39] and grouped into 4 categories: 1) managerial, or intellectual professions, 2) small to medium business owners or farmers, 3) manual, office or sales workers, and 4) unemployed. Finally, alcohol consumption was measured with a cut-off of 10 times or more per month, regular smoking was assessed with a cut-off of 11 cigarettes per day, and cannabis use was measured with a cut-off of 10 times or more per year [40]. These cut-off have been determined by the French Monitoring Center for Drugs and Drug Addictions or ”*Observatoire Français des Drogues et des Toxicomanies*” (OFDT).

### Statistical analyses

The prevalence rates for depression, suicidal ideations, suicide attempts and suicide risk severity were calculated by frequencies. Statistical analyses were performed separately for boys and girls on SAS V9.2. Chi-square tests were used to compare adolescent characteristics between suicide risk severity subgroups and family factors variables. Multivariate regressions were performed to assess the association between suicide risk severity and familial context variables adjusted on educational level, repeat school years, SES status, alcohol, tobacco, and cannabis use. A significant difference was considered to exist at p < 0.05. Odds Ratios were calculated with their 95% Confidence Interval.

## Results

### Socio-demographic characteristics, family factors and clinical data

The sample (n = 31,429) included 49.7% girls and 50.3% boys. The mean age was 17.4 years ±0.3. A large majority of the sample (98%) had followed classic or vocational school educational career at age 17. Around 44% had repeated a school year at least once. 7.2% of the parents were unemployed. Regarding family factors, 87.8% of the adolescents were living in their parents’ home and 12.2% of adolescents reported not living with their parents at age 17. In the entire sample, nearly 5% reported a negative relationship with their mother and 11.8% with their father. There were 24.4% of adolescents who had separated parents. When the parents were living together, 12.1% of the adolescents reported negative parental harmony.

Regarding substance use, we found that 7.8% of the adolescents were tobacco users, 8.9% were alcohol users and 13.5% were marijuana users. For depression, 7.5% of the adolescents had ADRS scores compatible with current depression (10.4% of the girls versus 4.5% of the boys, Chi-2 = 466, df =1, p < .001). Sixteen percent reported suicidal ideations (of whom 9.4% reported having suicidal ideations once and 6.8% reported having suicidal ideations several times) in the past 12 months*.* Eight percent reported lifetime suicide attempts (of whom 5.6% reported one suicide attempt and 2.7% several). The results are presented in Table 1.

**Table 1:** Socio-demographic characteristics, family factors and clinical data

		**Total**		**Girls**		**Boys**	
		**(N = 31429)**		**(N = 15087)**		**(N = 16342)**	
		**N**	**%**	**N**	**%**	**N**	**%**
**Socio-demographic characteristics**							
**Education**	Typical or vocational school	37817	97.9	18988	98.5	18829	97.37
	Out of school	799	2.1	290	1.5	509	2.63
**Grade repetition**	No	21894	55.6	11903	60.8	9991	50.5
	Yes	17467	44.4	7677	39.2	9790	49.5
**Parental occupation status**	Working	34767	92.8	17416	92.4	17351	93.2
	Unmployed	2702	7.2	1439	7.6	1263	6.8
**Family factors**							
**Not living at parent’s home**	Yes	4785	12.2	2306	11.9	2479	12.6
	No	34293	87.8	17136	88.1	17157	87.4
**Negative relationship with the mother**	Yes	1860	4.8	1039	5.3	821	4.2
	No	37232	95.2	18428	94.7	18804	95.8
**Negative relationship with the father**	Yes	4584	11.8	2656	13.8	1928	9.9
	No	34112	88.2	16598	86.2	17514	90.1
**Parental status and harmony**	Parents living together / positive relationship	22731	63.5	11074	61.8	11657	65.1
	Separated parents / negative relationship	6030	16.8	3149	17.6	2881	16.1
	Separated parents / positive relationship	2713	7.6	1228	6.9	1485	8.3
	Parents living together / negative relationship	4346	12.1	2457	13.7	1889	10.6
**Drug use**							
**Alcohol use**	No	35663	91.1	18729	96.0	16934	86.3
	Yes	3473	8.9	784	4.0	2689	13.7
**Tabacco use**	No	35856	92.2	18193	93.7	17663	90.8
	Yes	3023	7.8	1227	6.3	1796	9.2
**Marijuana use**	No	33917	86.5	17813	91.2	16104	81.9
	Yes	5290	13.5	1725	8.8	3565	18.1
**Depression and suicidal risk**							
**Depression**	No	34637	92.5	16903	89.6	17734	95.5
	Yes	2816	7.5	1970	10.4	846	4.5
**Suicidal ideations**	No	31847	83.8	15115	78.8	16732	89.0
	Yes	6151	16.2	4074	21.2	2077	11.0
**Suicidal attempts**	No	35090	91.8	16971	88.0	18119	95.6
	Yes	3146	8.2	2317	12.0	829	4.4
**Suicidal risk**	Grade 0	28672	91.2	13143	87.1	15529	95.0
	Grade 1	1049	3.3	676	4.5	373	2.3
	Grade 2	884	2.8	626	4.1	258	1.6
	Grade 3	824	2.7	642	4.3	182	1.1

### Suicide risk severity grade combining depression and suicidality

Three severity subgroups were defined: grade 1 (n = 1049, 3.4%) were depressed without suicidal ideations or attempts, grade 2 (n = 884, 2.8%) were depressed and reported suicidal ideations but no suicide attempts, and grade 3 (n = 824, 2.6%) were depressed and reported suicide attempts. The control group, grade 0, included 28,672 adolescents (91.2%) who were not depressed and had not reported suicide ideation in the past year or lifetime SA. The results are presented in Table 2.

**Table 2:** Risk severity grade combining depression and suicidality in girls and boys

**Girls**		**Grade 0**		**Grade 1**		**Grade 2**		**Grade 3**	
		**(N = 13143)**		**(N = 676)**		**(N = 626)**		**(N = 642)**	
		**N**	**%**	**N**	**%**	**N**	**%**	**N**	**%**
**Socio-demographic characteristics**									
**Education**	Out of school	137	1.1	.4	0.6	14	2.3	24	3.8
**Grade repetition**	Yes	4542	34.7	307	46.0	264	42.3	386	60.2
**Parental occupation status**	Unmployed	866	6.8	54	8.4	58	9.7	70	11.6
**Family factors**									
**Not living at parent’s home**	Yes	1327	10.2	77	11.6	82	13.3	98	15.5
**Negative relationship with the mother**	Yes	405	3.1	51	4.5	96	15.5	117	18.4
**Negative relationship with the father**	Yes	1325	10.3	125	19.0	151	24.4	196	31.2
**Parental status and harmony**	Parents living together with positive relationship	8074	66.7	334	54.6	277	47.8	236	41.5
	Separated parents with negative relationship	1838	15.2	114	18.6	127	21.9	147	25.8
	Separated parents with positive relationship	809	6.7	41	6.7	43	7.4	33	5.8
	Parents living together with negative relationship	1378	11.4	123	20.1	133	22.9	153	26.9
**Drug use**									
**Alcohol use**	Yes	395	3.0	28	4.2	34	5.5	112	9.0
**Tabacco use**	Yes	542	4.2	40	6.0	65	10.5	93	17.5
**Marijuana use**	Yes	878	6.7	67	10.0	77	12.4	134	20.9
**Boys**		**Grade 0**		**Grade 1**		**Grade 2**		**Grade 3**	
		**(N = 15529)**		**(N = 373)**		**(N = 258)**		**(N = 182)**	
		**N**	**%**	**N**	**%**	**N**	**%**	**N**	**%**
**Socio-demographic characteristics**									
**Education**	Out of school	315	2.1	11	3.0	.9	3.6	.8	4.5
**Grade repetition**	Yes	7211	46.6	228	61.6	114	44.5	113	62.4
**Parental occupation status**	Unmployed	928	6.3	34	10.0	20	8.4	.10	6.1
**Family factors**									
**Not living at parent’s home**	Yes	1814	11.8	41	11.1	43	16.8	47	26.5
**Negative relationship with the mother**	Yes	464	3.0	34	9.2	38	14.9	40	22.6
**Negative relationship with the father**	Yes	1228	8.1	57	15.8	59	23.3	56	31.8
**Parental status and harmony**	Parents living together with positive relationship	9574	67.7	172	54.3	106	47.1	77	50.0
	Separated parents with negative relationship	2104	14.9	63	19.9	52	23.1	40	25.6
	Separated parents with positive relationship	1173	8.3	22	6.9	15	6.7	.6	3.9
	Parents living together with negative relationship	1280	9.1	60	18.9	52	23.1	31	20.1
**Drug use**									
**Alcohol use**	Yes	1949	12.7	55	14.9	41	16.0	50	27.9
**Tabacco use**	Yes	1135	7.4	49	13.5	31	12.3	53	29.8
**Marijuana use**	Yes	2522	16.3	74	20.0	55	21.5	74	41.3

### Associations between family variables and severity grade of suicide risk adjusting for educational level, repeat school years, socio-economic status and substance use

**Figure 2:**
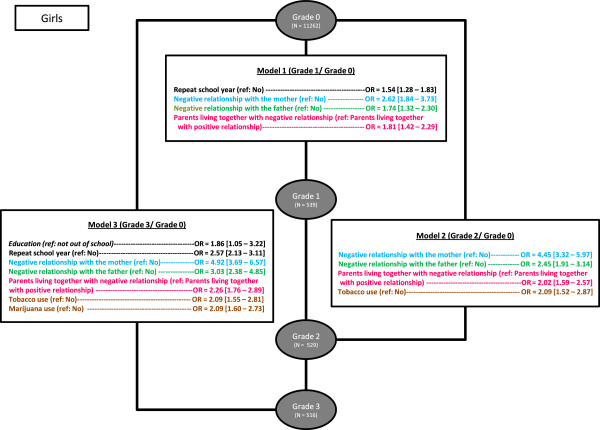
Associations between family variables and severity grade in girls adjusting for educational level, repeat school years, socio-economic status and substance use.

Associations between family variables, educational data, substance use and suicide risk severity grade combining depression and suicidality were assessed using multivariate analysis. Three severity subgroups were defined: grade 1 (depressed without suicidal ideations or attempts), grade 2 (depressed with suicidal ideations) and grade 3 (depressed with suicide attempts). The control group, grade 0, included adolescents not depressed and without suicidal ideations or attempts. We ran a series of multivariate logistic regression analyses to assess the association between suicide risk severity and family factors adjusted on educational level, repeated school years, SES status and substance use. In the model 1, the dependant variable was grade 1 versus grade 0, in the model 2: grade 2 versus grade 0 and in the model 3: grade 3 versus grade 0. Models were performed separately for boys and girls. Backward selection was used until all remaining variables had a *p* value <0.1. A significant difference was considered to exist at p < 0.05. Odds Ratios were calculated with their 95% Confidence Interval. The results are presented in Figures 2.

**Figure 3:**
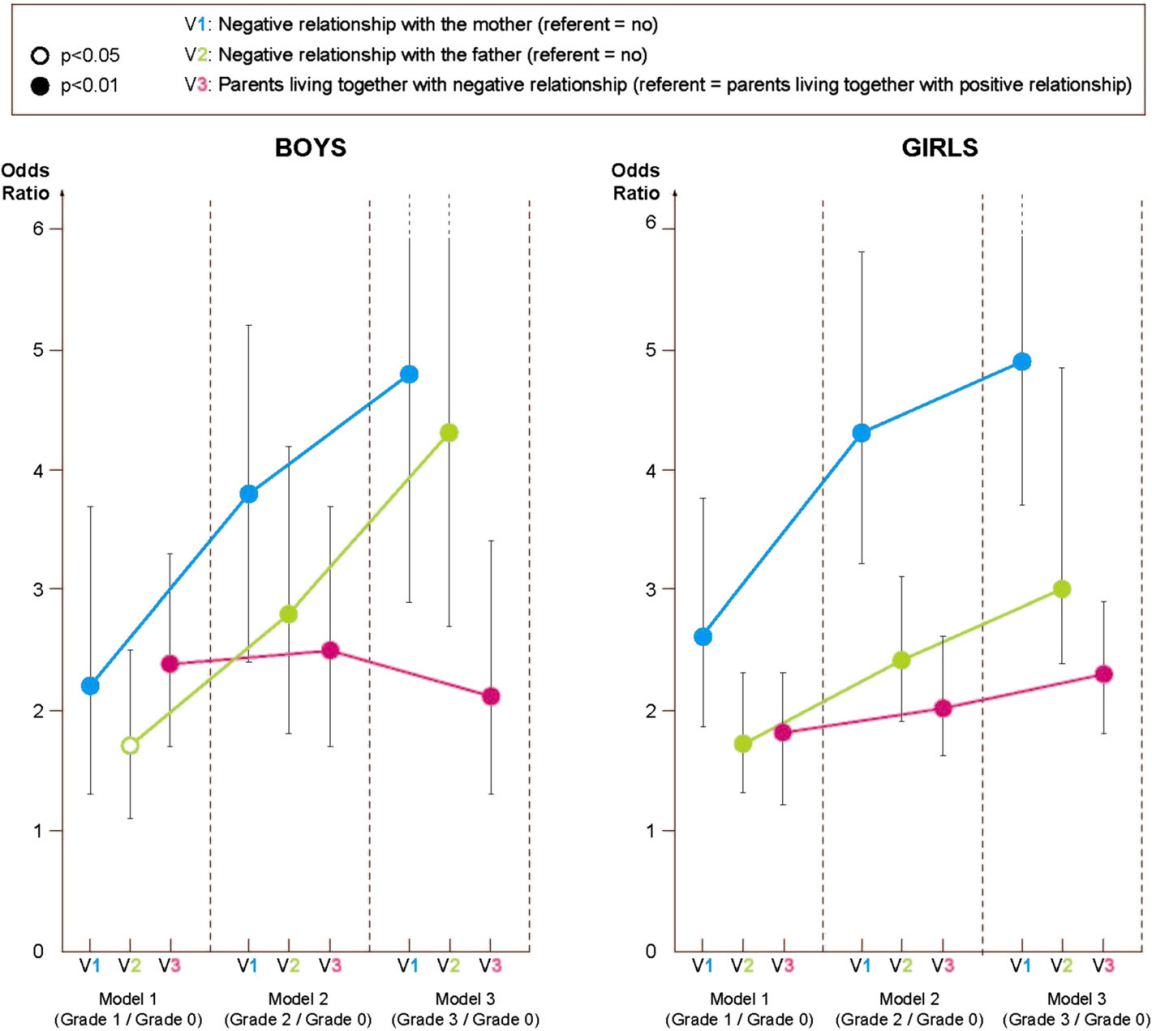
Associations between family variables and severity grade in girls and boys adjusting for confounding variables (graph).

Regarding girls (Figure 2), all substance use appeared to be associated with the severity grade combining depression and suicidality (grade 1 = depressed without suicidal ideation and without suicidal attempts, grade 2 = depressed with suicidal ideations and grade 3 = depressed with suicide attempts). Tobacco use reported by girls was associated with greater likelihood of belonging to risk severity grades 2 and 3 compared to controls (OR = 2.09 [1.55 – 2.81], p < 0.05 for both). Marijuana use was more likely to be associated with severity grade 3 compared to controls (OR = 2.09 [1.60 – 2.73], p < 0.05). Regarding educational data, repeat school years was associated with greater likelihood of risk severity grades 1 and 3 compared to the control group (grade 1: OR = 1.54 [1.28 – 1.83], p < 0.05 and grade 3: OR = 2.57 [2.13 – 3.11], p < 0.05). Regarding family variables, girls reporting a negative maternal relationship were more at risk for all severity grades compared to controls (grade 1: OR = 2.6 [1.84 – 3.73], p < 0.05, grade 2: OR = 4.4 [3.32 – 5.97], p < 0.05 and grade 3: OR = 4.9 [3.69 – 6.57], p < 0.05). Girls reporting a negative paternal relationship were also more at risk for all severity grades compared to controls (grade 1: OR = 1.7 [1.32 – 2.30], p < 0.05, grade 2: OR = 2.4 [1.91 – 3.14], p < 0.05 and grade 3: OR = 3 [2.38 – 4.85], p < 0.05). We also found that girls reporting that their parents were living together but in parental discord were more at risk for all severity grades compared to controls (grade 1: OR = 1.81 [1.42 – 2.29], p < 0.05, grade 2: OR = 2.02 [1.59 – 2.57], p < 0.05 and grade 3: OR = 2.26 [1.76 – 2.89], p < 0.05). The odds ratios for most family variables increased with severity (Figure 3). No significant statistical difference was found for girls reporting that their parents were divorced but did not have a negative relationship compared to controls.

**Figure 4:**
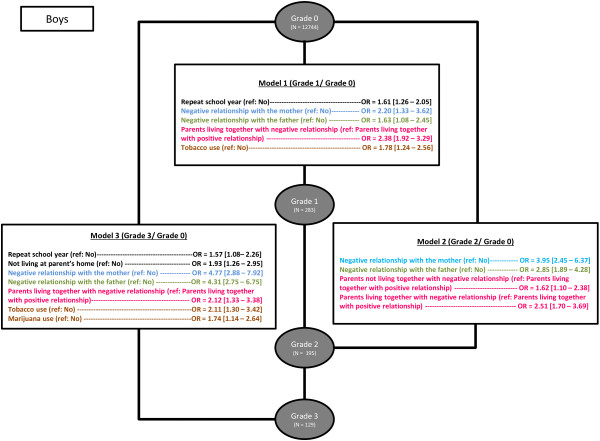
Associations between family variables and severity grade in boys adjusting for educational level, repeat school years, socio-economic status and substance use.

Results for boys (Figure 4) were very similar to those for girls. However, for boys two associations were slightly different regarding family factors. First, boys not living with their parents were significantly more likely to belong to grade 3 risk compared to controls (OR = 1.9 [1.26 – 2.95], p < 0.05). Second, having parents not living together and with a negative relationship was more associated with grade 2 risk compared to controls (OR = 1.6 [1.10 – 2.38], p < 0.05).

## Discussion

This study assessed the associations between depression, family factors and suicidality in a large representative community-based sample of adolescents aged 17 (n = 39,542), adjusting for confounding variables. Given data in the literature regarding depression as a proximal risk factor in suicidality [9] and the relevance of classifying suicidality (ideations and suicide attempt) [7], we divided the sample into 3 grades of suicide risk severity combining depression and suicidality (grade 1 = depressed without suicidal ideation and without suicide attempts, grade 2 = depressed with suicidal ideations and grade 3 = depressed with suicide attempts). The results confirmed previous risk factors for depression/suicidality in adolescents. Previously, school exclusion and academic difficulties have been implicated in suicidality in young people [17,41]. In France, given the high frequency of repeated years in school, this educational data also needs to be taken into account in assessment of suicidality among adolescents. All substance use including tobacco and marijuana use was associated with increased suicide risk in depressed adolescents. It has been shown that, unless comorbid, substance abuse disorders were not proximally associated with suicidality [9]. Adjusting on confounding variables (educational data, socio economic status, substance use), the results here showed that negative relationships with either or both parents, and parents’ living together with a negative relationship were significantly associated with depression and/or suicide risk in both genders (all risk severity grades) and that odds ratios increased according to risk severity grade. This means that what affects depression and suicidality is not parental separation per se, but rather parental harmony on the one hand, and perceived quality of the adolescents’ relationships with mother and father, on the other. Although we hypothesized different familial risk factors between girls and boys because of differential epidemiology, we found similar family risk factors in the two genders.

We found depression rates similar to those reported in the literature (e.g. the Center for Disease Control, for the year 2005–2006, found a depression prevalence between 4% and 6.4% in adolescents aged 12 to 17, without testing for gender differences ) [42]. We also had a higher prevalence in girls than boys, as found in many epidemiological studies [43-45]. Therefore, the higher prevalence of depression in girls than boys may not be a consequence of differential perceptions of family relationships. It should rather be interpreted as a consequence of other factors that were not assessed in the current study: e.g. genetic vulnerability, hormonal changes, gender specific social constraints, differential comorbid psychopathology [45-49].

The importance of family factors is strengthened by the fact that we found increases in odds ratios for most factors according to severity grade. Recent data suggested that defining and classifying suicidality could provide a better understanding of risk factors (proximal and distal) and interactions among them [7]. The recommended classification distinguishes depression, suicidal ideation and suicidal behavior in a hierarchical model [7,50]. Previous studies have underlined the role of family factors in suicidality in young people. First, adolescents who commit suicide are more likely to come from a family with a history of suicide and/or family psychopathology [17,19,20,51]. Second, childhood abuse, a history of separation and loss (by death or divorce) and exposure to physical and/or sexual violence are also associated with suicidality [16,52-55]. Third, adolescents with suicidal behaviors are more likely to be living in non-intact families [17,22,33,56-59] and their environment is characterized by problematic communication, poor attachment and high levels of conflict [14,16,17,28,29,34,51,57,60-62]. In depressed adolescents, poor family function is predictor of suicide attempts [1], and suicidal ideations and family conflict were independently associated with a suicidal event over a one-year follow-up [26]. Another recent study showed that the most common proximal risk factor for completed suicide for subjects younger than 30 years was conflict with family members, partners or friends [63]. Here, we focused on perceived intrafamilial relationships and found that negative relationship with either or both parents, and parents living together but with discord were significantly associated with suicide risk and/or depression in the two genders.

The current results have important clinical implications. Practitioners working with young people presenting depression and suicidal behaviors (ideation and/or attempts) should take the family factors into account, in particular aspects such as the adolescent’s relationships with either or both parents and relationships between parents whether or not they are living together. Assessing suicide risk in adolescents should include the assessment of family relationships and this could enable appropriate care to be provided for the adolescent and his family. A recent study assessed treatment of adolescent suicide attempters [64]. Depressed adolescents with prior suicide attempts were treated with a combination of medication and psychotherapy. After treatment, rates of improvement and remission of depression appeared comparable to those in non-suicidal depressed adolescents. The treatment included antidepressant medication and CBT (specifically developed to address suicide risk) including both individual and parent-adolescent sessions. Parent-adolescent sessions had probably contributed to this improvement. Of course, other psychotherapies have empirical evidences for its effectiveness such as family therapy.

The current study has several limitations. First, we could only focus on and assess a limited number of risk factors. Regarding adolescent psychopathology, 70 to 91% of young people who attempt or commit suicide present a psychiatric disorder [60,65]. Depression is the most common diagnosis in adolescents who commit suicide and it is highly prevalent in those with suicidal ideations and suicide attempts [15,65]. However, other conditions can interfere, but were not assessed in the current study (e.g. generalized anxiety disorder; disruptive behaviors; borderline personality disorder) [9,15,49,66,67]. Similarly, many non-clinical risk factors were not assessed (e.g. life stressors, problems with authorities, relationship problems with peers, sexual and physical abuse, low socio-economic status) [7]. Second, as our study was cross sectional, meaning that the assessment of suicidal behaviors and changes in family structure was retrospective and that the mechanisms underpinning the associations could not be investigated. Only longitudinal studies are able to explore the different effects of the potential moderators of associations. Third, we had no data available on ethnicity because in France it is not allowed by ethics committees. It can however be noted that the present data only concerned French people from metropolitan France (i.e. excluding overseas territories). The sample nevertheless included 5% of the French metropolitan population aged 17 and was representative of it. Fourth, we had a differential temporal focus for our clinical variables. Current depression was measured for the previous 2 weeks, suicide ideations concerned the past 12 months and suicide attempts concerned lifetime. However, (1) given that subjects were 17 years old, suicide attempts mostly concerned the previous 5 years; (2) prior suicide attempt is an important risk factor for suicidality in young people. In addition, we did not differentiate single suicide attempt and lifetime history of several attempts because of the small numbers of subjects in each subgroup. Thus, grade 3 risk severity included adolescents with a history of one or several suicide attempts. Finally, our aim to investigate current depression as a proximal risk factor led us to exclude many adolescents who had experienced suicidal ideations in the past 12 months and/or lifetime suicide attempt(s) but were not depressed at the time of assessment (see Figure 1). The same analyses (multivariate analysis) as those conducted in this study were performed on the excluded sample and showed similar results for family risk factor (see Additional file 1: Figure S1). Therefore the exclusion of these subjects did not radically modify our results. Finally, self-report of family functioning was also a limitation because depression may lead to a negative perception bias regarding relationships with parents.

The study also has some strength. First, the study included a large representative population-based sample of French adolescents aged 17 which allowed an exhaustive investigation of suicide risk severity in depressed adolescents. In addition, the setting in which the study was implemented (JAPD) was a good guarantee of methodological thoroughness for sampling and conditions of administration. Compared to studies conducted in adult populations, we were able to restrict recall bias because subjects were all 17 years old. Second, depression assessment was performed on a scale specific to adolescents [38]. In previous studies, depression has often been lifetime depression so that it was difficult to know if depression reported by a subject was present before, during or after suicide attempts. Third, results regarding family factors were adjusted on several confounding variables (educational level, repeat school years, socio-economic status, and substance use). Fourth, because of the good statistical power, we were able to (1) run multivariate analyses on each gender; (2) distinguish family separation, family discord and perceived parental relationship.

## Conclusion

This study has provided keys regarding the epidemiology of suicidal behaviours in a large community-based sample of French adolescents aged 17. Substance use, repeat school years and family factors were associated with an increased suicide risk in depressed adolescents, with no difference according to gender. Specifically, perceived negative relationships with either or both parents, and a negative relationship between parents, whether living together or not, were associated with suicidality. So it appears essential to take into account real and perceived intrafamilial relationships among depressed adolescents to assess and prevent suicidal behaviours.

## Competing interests

Dr Consoli reported receiving travel support from Bristol-Myers Squibb and Dr. Cohen reported past consultation for or the receipt of honoraria from Schering-Plough, Bristol-Myers Squibb, Otsuka, Janssen, Shire, and Sanofi-Aventis. Dr Revah-Levy, Moro, Peyre, Speranza, Falissard, Mme Hassler, Touchette have no relationships that might have interest in the submitted work. No authors have any non-financial interests that may be relevant to the submitted work.

## Authors’ contributions

Study concept and design: ARL, ET, AC, HP, BF. Acquisition of data: CH, ARL, HP, ET. Statistical analysis: BF, DC, HP, CH. Interpretation of data: all authors. Drafting the manuscript: AC, ARL, DC. Critical revision of the manuscript revision for important intellectual content: MRM, ARL, MS, DC, BF. Final draft: all authors. All authors read and approved the final manuscript.

## Supplementary Material

Additional file 1: Figure S1 Excluded subgroup. Click here for file
