# Supplementary material for: Suicidal behaviors in depressed adolescents: role of perceived relationships in the family
Source: Child Adolesc Psychiatry Ment Health. 2013 Mar 16;7:8. doi: 10.1186/1753-2000-7-8 (PMC3655930; doi:10.1186/1753-2000-7-8)
Supplement: Additional file 1: Figure S1 — Excluded subgroup. [file 1753-2000-7-8-S1.ppt]

## Slide 1
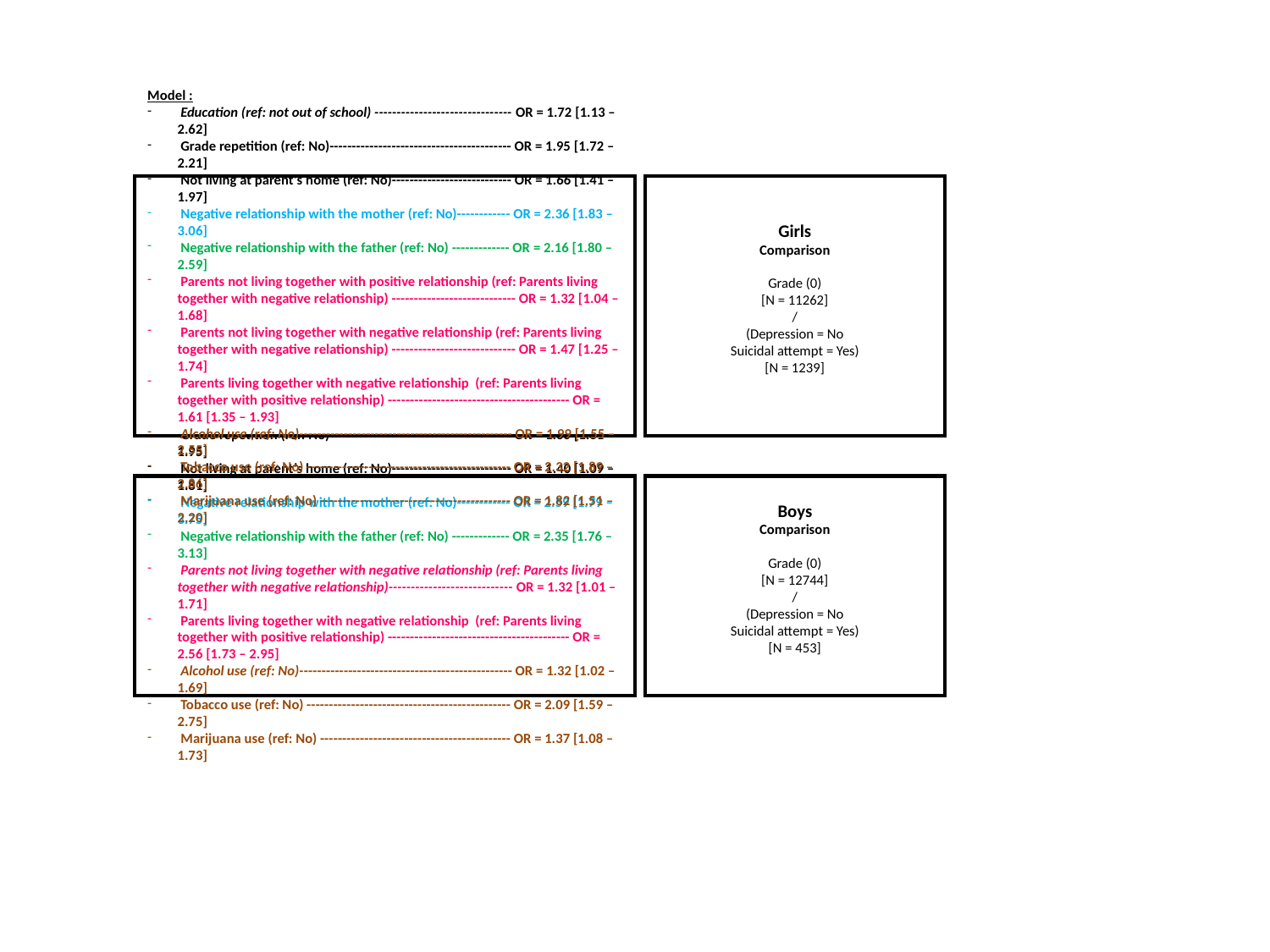

Model :
 Education (ref: not out of school) ------------------------------- OR = 1.72 [1.13 – 2.62]
 Grade repetition (ref: No)----------------------------------------- OR = 1.95 [1.72 – 2.21]
 Not living at parent’s home (ref: No)--------------------------- OR = 1.66 [1.41 – 1.97]
 Negative relationship with the mother (ref: No)------------ OR = 2.36 [1.83 – 3.06]
 Negative relationship with the father (ref: No) ------------- OR = 2.16 [1.80 – 2.59]
 Parents not living together with positive relationship (ref: Parents living together with negative relationship) ---------------------------- OR = 1.32 [1.04 – 1.68]
 Parents not living together with negative relationship (ref: Parents living together with negative relationship) ---------------------------- OR = 1.47 [1.25 – 1.74]
 Parents living together with negative relationship (ref: Parents living together with positive relationship) ----------------------------------------- OR = 1.61 [1.35 – 1.93]
 Alcohol use (ref: No)------------------------------------------------ OR = 1.99 [1.55 – 2.55]
 Tobacco use (ref: No) ---------------------------------------------- OR = 2.32 [1.89 – 2.86]
 Marijuana use (ref: No) ------------------------------------------- OR = 1.82 [1.51 – 2.20]
Girls
Comparison
Grade (0)
[N = 11262]
/
(Depression = No
Suicidal attempt = Yes)
[N = 1239]
Model :
 Grade repetition (ref: No)----------------------------------------- OR = 1.60 [1.31 – 1.95]
 Not living at parent’s home (ref: No)--------------------------- OR = 1.40 [1.09 – 1.81]
 Negative relationship with the mother (ref: No)------------ OR = 2.59 [1.79 – 3.75]
 Negative relationship with the father (ref: No) ------------- OR = 2.35 [1.76 – 3.13]
 Parents not living together with negative relationship (ref: Parents living together with negative relationship)---------------------------- OR = 1.32 [1.01 – 1.71]
 Parents living together with negative relationship (ref: Parents living together with positive relationship) ----------------------------------------- OR = 2.56 [1.73 – 2.95]
 Alcohol use (ref: No)------------------------------------------------ OR = 1.32 [1.02 – 1.69]
 Tobacco use (ref: No) ---------------------------------------------- OR = 2.09 [1.59 – 2.75]
 Marijuana use (ref: No) ------------------------------------------- OR = 1.37 [1.08 – 1.73]
Boys
Comparison
Grade (0)
[N = 12744]
/
(Depression = No
Suicidal attempt = Yes)
[N = 453]
